# Transcriptome analysis of five different tissues of bitter gourd (*Momordica charantia* L.) fruit identifies full-length genes involved in seed oil biosynthesis

**DOI:** 10.1038/s41598-022-19686-4

**Published:** 2022-09-13

**Authors:** Kumar Ravichandiran, Madasamy Parani

**Affiliations:** Department of Genetic Engineering, College of Engineering and Technology, Faculty of Engineering and Technology, SRM Institute of Science and Technology, SRM Nagar, Kattankulathur, Chengalpattu, Tamil Nadu 603203 India

**Keywords:** Genomics, Plant genetics, Sequencing

## Abstract

The bitter gourd seed oil, rich in conjugated fatty acids, has therapeutic value to treat cancer, obesity, and aging. It also has an industrial application as a drying agent. Despite its significance, genomics studies are limited, and the genes for seed oil biosynthesis are not fully understood. In this study, we assembled the fruit transcriptome of bitter gourd using 254.5 million reads (Phred score > 30) from the green rind, white rind, pulp, immature seeds, and mature seeds. It consisted of 125,566 transcripts with N50 value 2,751 bp, mean length 960 bp, and 84% completeness. Transcript assembly was validated by RT-PCR and qRT-PCR analysis of a few selected transcripts. The transcripts were annotated against the NCBI non-redundant database using the BLASTX tool (E-value < 1E−05). In gene ontology terms, 99,443, 86,681, and 82,954 transcripts were classified under biological process, molecular function, and cellular component. From the fruit transcriptome, we identified 26, 3, and 10 full-length genes coding for all the enzymes required for synthesizing fatty acids, conjugated fatty acids, and triacylglycerol. The transcriptome, transcripts with tissue-specific expression patterns, and the full-length identified from this study will serve as an important genomics resource for this important medicinal plant.

## Introduction

Bitter gourd (Momordica charantia L.) is a vegetable crop widely cultivated in Asia and Africa. It is also called bitter melon, balsam-apple, bitter apple, balsam pear, balsam birne, African cucumber, bitter cucumber, bittergurke, karela, and carilla gourd^1^. It is a climber belonging to the Cucurbitaceae family and has about 60 annual and perennial species^2,3^. The bitter gourd genome is made of 2n = 2x = 22 chromosomes and 339 Mb. Bitter gourd fruits and leaves have a long history of use in traditional medicine for treating diabetes, infection, wounds, and osteoarthritis^4^. It can be used for birth control, abortion, and treating ailments like jaundice, leprosy, piles, psoriasis, and rheumatism^5,6^. In a randomized controlled trial, oral intake of dried bitter gourd fruit pulp 2000 mg/day exhibited hypoglycemic effect as indicated by a significant reduction in fructosamine levels^7^. In another clinical trial, daily consumption of 2.5 g of dried whole bitter gourd fruit powder (equivalent to 50 g fresh bitter gourd fruit) significantly lowered the blood glucose levels in individuals with prediabetes. The glucose lowering effect was higher among the participants with higher baseline fasting plasma glucose (FPG) levels^8^. A randomized controlled trial in type-2 diabetes patient without other complications showed that supplementation of bitter gourd fruit powder along with Metformin and Glibenclamide provided improved glycaemic control (*p* < 0.05)^9^.

Besides the fruits and leaves, bitter gourd seed oil (BGSO) also has therapeutic and industrial applications. The BGSO content in different bitter gourd varieties varies between 28 and 30%^10,11^. It is a rich source of polyunsaturated fatty acids and is considered a special oil due to the presence of 56–62% ⍺-eleostearic acid^10,12^. BGSO is used to manufacture paints, inks, and other coating materials because of its excellent drying property^13^. BGSO has significant health benefits because of its use in treating diabetes, cancer, obesity, and aging^14–17^. These properties were supported by mechanistic studies in the cell lines and animal models. Treatment with 10–100 mg/Kg body weight BGSO significantly reduced edema, epithelial disruption, mucosa erosions, and ulceration in rats. The gastric juice of the treated rats showed elevated the pH without decreasing total acidity^18^. Accumulation of intracellular triglycerides was significantly lower in the hepatoma cells treated with BGSO than those treated with α-linolenic acid. Anti-lipidemic property of BGSO was confirmed in vivo in mice, and it was attributed to an increase in NAD + /NADH ratio and activation of PPAR⍺, AMPK, and SIRT1 pathways^19^. BGSO showed anti-adiposity effect in mice, as indicated by the fat and plasma leptin content^20^. BGSO exhibited hypoglycemic potential by inhibiting α-glucosidase and α-amylase by 53% and 38%, respectively^21^. Oral diet supplemented with 0.5% BGSO nanoemulsion conferred protection against oxidative stress in the experimentally induced diabetic rats^22^.

Anti-cancer property of BGSO is supported by several experiments conducted in vitro and in vivo. BGSO triggered apoptosis in the colon cancer cells by upregulating GADD45 and p53 and downregulating Bcl-2 expression^23^. It was also reported that α-eleostearic acid and its dihydroxy derivatives from BGSO inhibited the growth of leukemia and colon carcinoma cell lines^24^. In breast cancer cell lines, α-eleostearic acid showed anti-cancer activity by arresting the cell cycle and decreasing HER2/HER3 levels^25^. It was observed that α-eleostearic acid inhibited tumor formation and metastasis in the triple-negative breast cancer mice model^26^. Oral supplementation of hepatocellular carcinoma-induced rats with BGSO reduced hepatic dysplastic nodules and neoplastic lesions^27^.

Despite the significance of bitter gourd in therapeutic and industrial applications, research on large-scale genomic studies and cloning of the genes involved in oil biosynthesis is limited. RNA-Sequencing is a cost-effective and high-throughput method to study genes and their expression patterns in qualitative and quantitative aspects. It also helps understand the functional elements of the genome and identify the genes involved in various pathways^28,29^. In the current study, we analyzed the bitter gourd fruit transcriptome in detail and identified full-length genes coding for all the enzymes involved in the biosynthesis of BGSO.

## Results and discussion

### Assembly of bitter gourd fruit transcriptome

**Figure 1:**
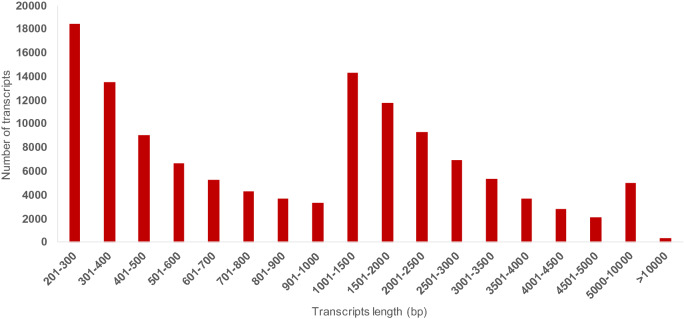
Length distribution of the assembled transcripts from bitter gourd fruit transcriptome.

We generated 295.9 million 150 bp raw reads from the green rind, white rind, pulp, immature seeds, and mature seeds of bitter gourd. The reads from individual tissues varied between 33.3 million (green rind) and 84.2 million (immature seeds). Post quality filtering for low-quality reads (Phred score < 30) and adaptor removal, 254.5 million reads (86%) were retained for further processing (Table 1). About 14% of the reads were dis-carded after quality filtering. Even though the rejection rate is higher than the average percentage of data discarded after quality filtering, the total number of reads available for assembly is much higher than the requirement for a good assembly. Using trinity assembler, assembly of these reads generated 146,719 transcripts with 1,064 bp mean length and 2,855 bp N50. After clustering using CD-HIT, we obtained 125,566 transcripts with 960 bp mean length and 2,751 bp N50 (Table 2). The length distribution of the assembled transcripts is given in Fig. 1. The assembly parameters indicated that the transcripts assembled in this study could be reliably used for gene prediction and other downstream analyses.

**Table 1:** Summary of raw reads and Q30 reads obtained from different tissues of bitter gourd.

S. No	Sample name	Number of reads (million)	Number of bases (Gb)	Q30 >	
				Number of reads (million)	Number of bases (Gb)
1	Green rind	33.3	4.8	28.1	3.3
2	White rind	51.0	7.4	43.8	5.2
3	Seed	52.6	7.5	45.7	5.5
4	Pulp	74.9	10.8	64.0	7.6
5	Immature seeds	84.2	12.1	72.9	8.5
	Total	295.9	42.6	254.5	30.1

**Table 2:** De novo assembly statistics of bitter gourd fruit transcriptome.

Particulars	Number
Number of raw reads (in million)	295.9
Number of clean reads (in million)	254.5
No of bases (after processing in Gb)	30.1
Mean Phred Score	30
Total transcripts	125,566
Total length (bases in million)	196.86
Average length (bases)	1567.86
Median contig length	960
GC (%)	40.61
Contig N50 (bases)	2,751

### Annotation of the transcripts

**Figure 2:**
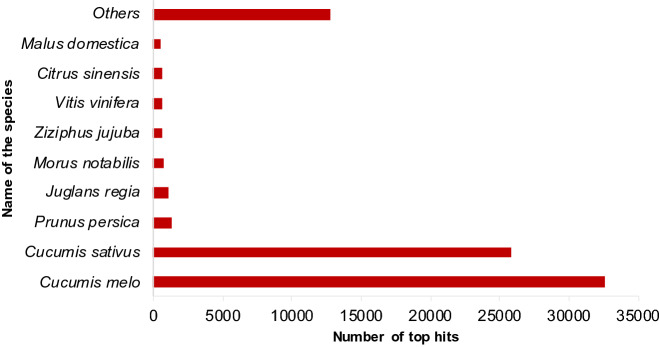
BLASTX top hit species distribution of bitter gourd transcripts against the nr database.

Based on BLASTX similarity searches, we annotated 76,794 transcripts, and 48,771 transcripts did not show significant similarity with the existing genes. The BLASTX results were analyzed using BLAST2GO for further annotation. As shown in Fig. 2, the top-hit species for the bitter gourd transcripts was *Cucumis mel*o followed by *Cucumis sativus*. According to the APG IV phylogenetic classification^30^, both the top-hit species are taxonomically closely related to bitter gourd.

### Functional classification of the transcripts

**Figure 3:**
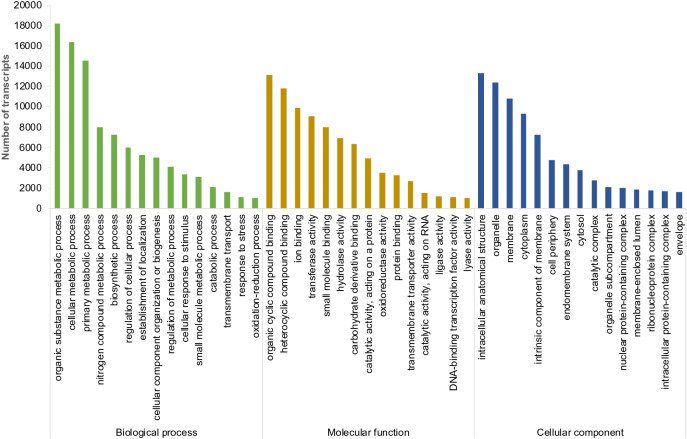
Gene Ontology classification of bitter gourd transcripts under three major categories of GO terms.

The functional classification of the transcripts based on GO terms showed that 99,443 and 86,681, and 82,954 were classified under biological process, molecular function, and cellular component, respectively. Under the biological process, the highest number of the transcripts were related to the metabolic process (18,231 transcripts), followed by the cellular metabolic process (16,342 transcripts) and primary metabolic process (14,567 transcripts). In the case of molecular function, transcripts involved in organic cyclic compound binding were more abundant (13,119 transcripts), followed by heterocyclic compound binding (11,765 transcripts) and ion binding (9,878 transcripts). In the cellular component category, transcripts responsible for the intracellular anatomical structure were found in more numbers (13,265 transcripts) than organelle (12,412 transcripts), membrane (10,768 transcripts), and others (Fig. 3).

### Quantification and completeness analysis of the transcripts

The abundance of the transcripts in the transcriptome was calculated based on the FPKM value using RSEM. The data for all the transcripts from the respective tissues are given in Supplementary Table S3. The top 15 abundant transcripts in each tissue and overall abundance in the fruit transcriptome are given in Supplementary Table S4. The results showed that some genes are abundantly expressed in each tissue. The observed expression patterns of the transcripts can be used to isolate tissue-specific genes and promoters for applied research in bitter gourd. For example, the proteins from the bitter gourd fruit pulp showed antidiabetic properties in vitro and in vivo^31,32^ but the corresponding genes are yet to be cloned. Analysis of gene completeness showed 94% complete BUSCOs (Supplementary Fig. S1), which indicated that the transcript assembly is of good quality.

### Validation of transcript assembly

**Figure 4:**
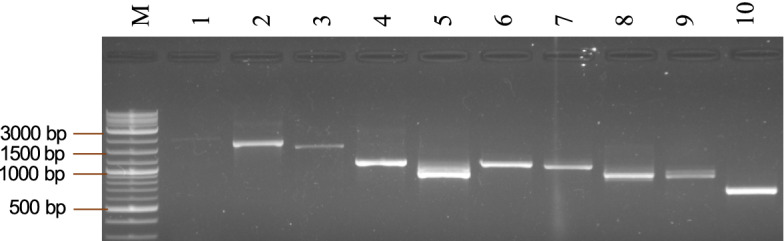
Reverse transcription PCR amplification of ten selected genes from bitter gourd. M is the 1 Kb DNA marker, followed by the RT-PCR product from Squalene synthase (lane 1), Hydroxymethylglutaryl-CoA synthase (lane 2) Acetyl-CoA acetyltransferase (lane 3), Methyltransferase-like protein 13 (lane 4), Phytoene synthase (lane 5), 3-hydroxy-3-methylglutaryl-coenzyme A reductase (lane 6), Chaperone protein dnaJ 10 (lane 7), Isopentenyl phosphate kinase (lane 8), Myb-related protein 306 (lane 9), and Late embryogenesis abundant protein M17 (lane 10).

The Assembly of the transcripts was validated by performing reverse transcription PCR (RT-PCR) amplification of selected genes using the RNA from the same tissues. We selected ten transcripts ranging in size between 609 bp and 2,567 bp, and their details are given in Supplementary Table S1. In agarose gel analysis of the RT-PCR products, we observed the amplification of cDNA fragments of approximately the same size as expected based on the transcript assembly (Fig. 4). This result indicated that the transcripts assembled in this study could be reliably used for gene identification.

### Validation of gene expression

**Figure 5:**
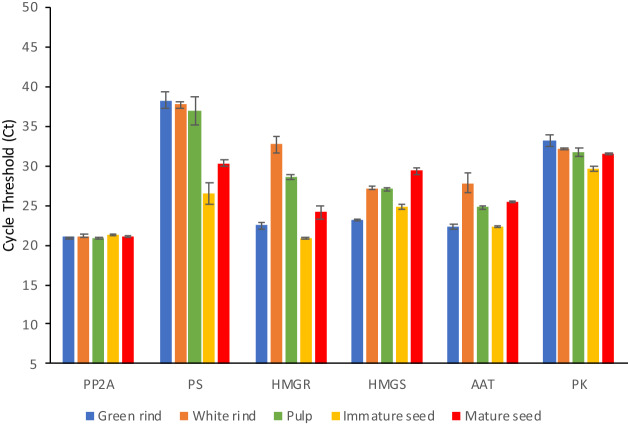
Quantitative reverse transcription PCR (qRT-PCR) analysis of Serine/Threonine-protein phosphatase (PP2A), Phytoene synthase (PS), 3-Hydroxy 3-Methylglutaryl CoA reductase (HMGR), Hydroxymethyl glutaryl CoA synthase (HMGS), Acetyl CoA acetyltransferase (AAT), and Phosphate kinase (PK).

The expression of selected transcripts associated with terpenoid and steroid bio-synthesis pathways was validated using qRT-PCR. The genes chosen for this study included Phytoene synthase, Acetyl-CoA transferase, 3-Hydroxy-3-methylglutaryl-coenzyme A reductase 1, Phosphate kinase, and Hy-droxymethylglutaryl-CoA synthase. The FPKM values for these genes are given in Supplementary Table S5. Expression of these genes, as assessed from the cycle threshold (ct) values, largely correlated with the expression levels in terms of FPKM (Fig. 5).

### Identification of simple sequence repeats (SSRs)

**Figure 6:**
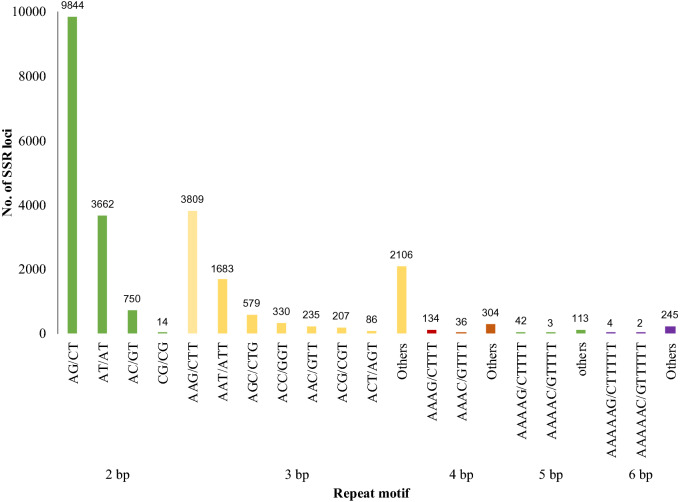
Frequency of different types of SSR motifs identified from bitter gourd fruit transcriptome.

We identified 57,093 SSRs from 125,566 assembled transcripts. The majority of the SSRs were either di-nucleotide repeats (59.0%) or tri-nucleotide repeats (37.4%). Di-nucleotide repeats with six repeats and tri-nucleotide repeats with five repeats were found to be the most abundant ones. Only a small proportion of the SSRs belonged to the tetra-nucleotide (2.0%), hexa-nucleotide (1.0%), and penta-nucleotide repeats (0.65%) (Table 3). AG/CT was the most abundant type (68.98%) in terms of the actual sequence present in the repeats. AAG/CTT was the most abundant tri-nucleotide repeat (42.16%). The details of different types of repeats identified from the transcripts are given in Fig. 6. SSR marker-based study of genetic diversity in 211 accessions of bitter gourd showed clear segregation according to geographical origin^33^. Alhariri et al.^34^ have analyzed 51 accessions using 61 primers and clustered the accession based on the polymorphism in SSR markers. Surprisingly, the 51 accessions formed three groups according to fruit size. These reports indicate that the SSR markers identified in this study will be highly useful for varietal identifications, mapping, and association studies in bitter gourd.

**Table 3:** Type of SSR repeats and their frequency identified from bitter gourd fruit transcriptome.

Motif length	Repeats								
	5	6	7	8	9	10	> 10	Total	%
Di	0	5374	2785	1855	1104	698	2454	14,270	59.00
Tri	4617	2145	1001	686	108	140	338	9035	37.35
Tetra	328	109	17	17	3	0	0	474	1.96
Penta	127	27	1	1	0	2	0	158	0.65
Hexa	143	71	15	11	6	5		251	1.04
Total	5215	7726	3819	2570	1221	845	2792	24,188	100
%	21.56	31.94	15.79	10.63	5.05	3.49	11.54

### Identification of transcripts related to secondary metabolites

**Figure 7:**
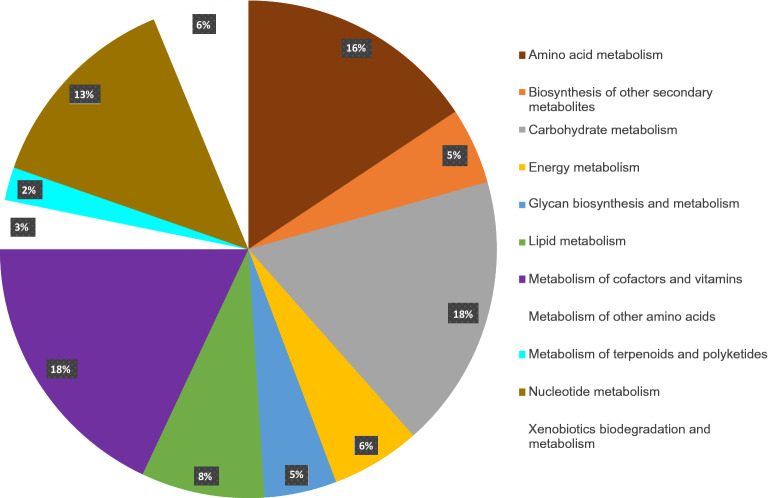
KEGG pathway analysis of the bitter gourd fruit transcriptome.

In KEGG pathway analysis, we mapped 25,497 transcripts to 147 pathways. About half of the mapped transcripts (51.5%) were related to the metabolism of cofactors and vitamins (18%), carbohydrates (17.8%), amino acids (15.7%) (Fig. 7). Antidiabetic properties of bitter gourd fruits have been demonstrated in several in vitro, in vivo, and clinical studies^8,35,36^. Most of the compounds responsible for its bitter taste and antidiabetic properties are triterpenoids and steroids^37^. The carbon skeletons of cucurbitacin triterpenoids are derived from 2,3-oxidosqualene by the action of oxidosqualene cyclase. Although cucurbitacins accumulated in high amounts in the fruits, cucurbitadienol synthase gene was highly expressed in the leaves. This indicated that cucurbitacins might be synthesized in the leaves and transported to the fruits^38^. Charantin also accumulated in high amounts in the fruits compared to the root, stem, leaves, and flowers^39^. The same study identified fifteen genes related to triterpenoid biosynthesis from the RNASeq library constructed from the bitter gourd seedlings. However, most of them encoded for the enzymes involved only up to the synthesis of squalene and 2,3-oxidosqualene. We identified 191 and 99 transcripts related to the terpenoid backbone and steroid biosynthesis from the bitter gourd fruit transcriptome. In our study also, most of the transcripts were mapped up to the synthesis of squalene and squalene-2,3-epoxide (Supplementary Figures S2 & S3). We could not identify the genes involved in the synthesis of cucurbitacin and charantin. Together with previous reports, our results indicate that mature leaves may be a good source for cloning the genes related to the biosynthesis of the specific triterpenoid and steroid compounds from bitter gourd.

### Identification of full-length genes related to bitter gourd seed oil biosynthesis

**Figure 8:**
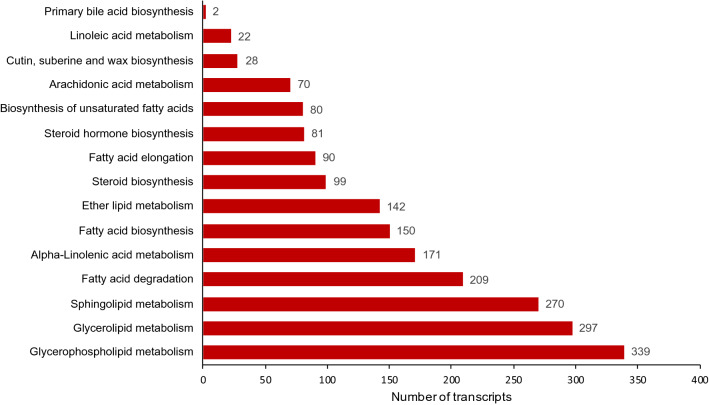
Details of the number of transcripts involved in lipid metabolism in the bitter gourd fruit transcriptome.

**Figure 9:**
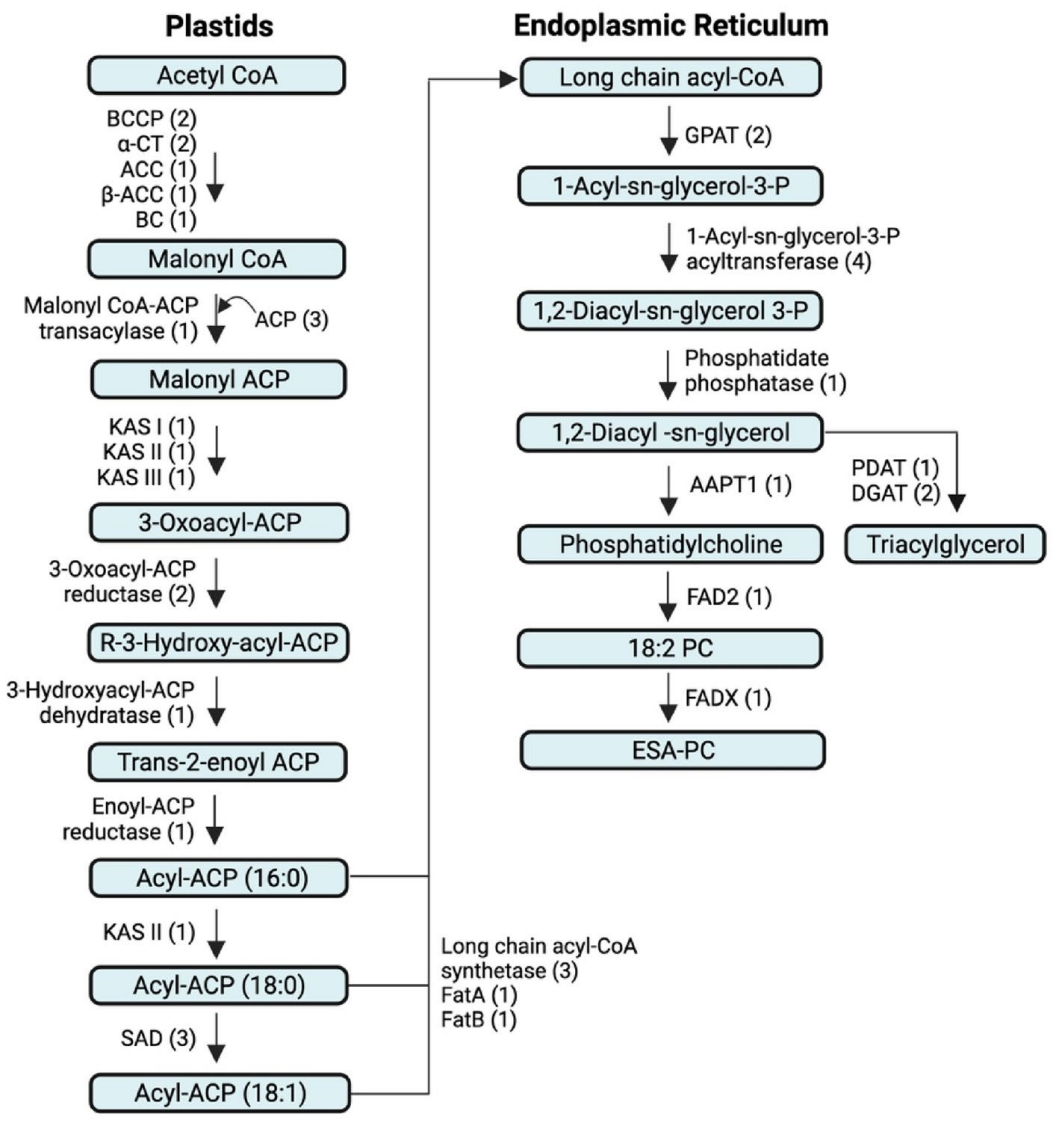
Schematic representation of the biochemical pathway for synthesizing fatty acids, conjugated fatty acids, and triacylglycerol. The numbers given in the bracket indicate the number of full-length genes identified for the respective enzymes and carrier proteins from the bitter gourd fruit transcriptome. *BCCP* biotin carboxyl carrier protein of acetyl-CoA carboxylase; *⍺-CT* acetyl-CoA carboxylase carboxyl transferase subunit alpha; *ACC* acetyl-CoA carboxylase; *β-ACC* acetyl-CoA carboxylase beta subunit; *BC* biotin carboxylase; *ACP* acyl carrier protein; *KAS I* 3-oxoacyl-ACP synthase; *KAS II* 3-oxoacyl-ACP synthase II; KAS *III* 3-oxoacyl-ACP synthase III; *SAD* stearoyl-ACP-desaturase; *FatA* oleoyl-ACP thioesterase; *FatB* acyl-ACP thioesterase ATL3; *GPAT* glycerol-3-phosphate acyltransferase; *AAPT* phosphatidylcholine:diacylglycerol cholinephosphotransferase; *FAD2* delta(12)-fatty acid desaturase 2; *FADX* delta(12)-fatty acid conjugase; *PDAT* phospholipid:diacylglycerol acyltransferase; *DGAT* diacylglycerol O-acyltransferase; *PC* phosphatidylcholine; *ESA* eleostearic acid.

We mapped 2,050 transcripts to lipid metabolism from the assembled transcripts, including 15 pathways related to the biosynthesis and metabolism of various fatty acids and lipids (Fig. 8). Among them, 150, 80, 297, and 339 transcripts were related to the biosynthesis of fatty acids, unsaturated fatty acids, glycerolipids, and glycerophospho-lipids. Plants synthesize various fatty acids and lipids, which have medicinal, cosmetic, and industrial applications^40–42^. Fatty acid biosynthesis occurs in plastids using acetyl Co-A as the major precursor and involves fatty acid synthases and carrier proteins. Fatty acids are synthesized up to 16:0 long-chain acyl carrier protein (ACP) during this process. The 16:0 fatty acids are elongated to 18:0 ACP and desaturated to 18:1 ACP by ketoacyl synthase II (KAS II) and stearoyl-ACP desaturase, respectively. All the long-chain ACPs from the chloroplast contribute to the long-chain acyl-CoA pool, which is used to syn-thesize oils and lipids in the endoplasmic reticulum. Fatty acids are incorporated into glycerol-3P to obtain diacylglycerol (DAG). The DAG pool is used to make triacylglycerol (oil) or conjugated fatty acids through distinct pathways. A schematic diagram representing the biochemical pathways of fatty acids, conjugated fatty acids, and triacylglycerol is shown in Fig. 9.

Yang et al. 2010 studied the transcriptome of bitter gourd seeds by 454 sequencing and identified several transcripts related to oil biosynthesis genes. However, full-length genes were analyzed only for diacylglycerol acyltransferase 1 and 2^43^. In our study, we identified 2,050 transcripts related to lipid metabolism. The transcripts related to fatty acid biosynthesis, glycerolipid metabolism, and glycerophospholipid metabolism were analyzed to determine full-length genes coding for the enzymes and proteins involved in the biosynthesis of fatty acids conjugated fatty acids, and triacylglycerol. We identified 39 full-length genes (GenBank Accession No: ON175892 to ON175930), which code for 23 enzymes and two carrier proteins required for the biosynthesis of fatty acids, conjugated fatty acids, and triacylglycerol (Fig. 9, Table 4). This included ten full-length genes coding for the five enzymes needed for converting long-chain acyl-CoA to triacylglycerol. The highest number of four genes were identified for 1-acyl-sn-glycerol-3-phosphate acyltransferase (AGPAT). We also found two genes for diacylglycerol acyltransferase (DGAT) and one for phospholipid: diacylglycerol acyltransferase (PDAT), which are essential to convert diacyl glycerol pool to triacylglycerol. These oil biosynthesis genes can be used to increase the oil content through genetic engineering approaches. This is evident from the studies, which showed that overexpression of heterologous genes could achieve as high as a ten-fold increase in seed oil content^44,45^.

**Table 4:** Details of the full-length genes identified from the bitter gourd fruit transcriptome.

S. no	Name of the gene	mRNA (nt)	CDS (bp)	ORF (aa)	5ʹUTR (bp)	3ʹUTR (bp)	Genbabank accession no
1	Biotin carboxyl carrier protein of acetyl-CoA carboxylase (BCCP), chloroplastic	1484	867	288	398	219	ON175892
2	Biotin carboxyl carrier protein of acetyl-CoA carboxylase 1, (BCCP) chloroplastic	1419	852	283	119	448	ON175893
3	Acetyl-CoA carboxylase carboxyl transferase subunit alpha (⍺-CT), chloroplastic isoform 1	2641	2205	734	277	159	ON175894
4	Acetyl-CoA carboxylase carboxyl transferase subunit alpha (⍺-CT), chloroplastic isoform 2	3508	2367	788	350	791	ON175895
5	Acetyl-CoA carboxylase 1 (ACC)	8184	6798	2265	957	429	ON175896
6	Acetyl-CoA carboxylase beta subunit (β-ACC)	3965	1578	525	216	2171	ON175897
7	Biotin carboxylase 1 (BC 1), chloroplastic	2070	1590	529	143	337	ON175898
8	Malonyl CoA-ACP transacylase (MCAT)	2000	1137	378	449	414	ON175899
9	Acyl carrier protein (ACP)	803	462	153	168	173	ON175900
10	Acyl carrier protein 1(ACP 1), chloroplastic	765	420	139	121	224	ON175901
11	Acyl carrier protein 4 (ACP 4), chloroplastic	1614	432	143	305	877	ON175902
12	3-oxoacyl-ACP synthase I (KAS I), chloroplastic	2172	1413	470	331	428	ON175903
13	3-oxoacyl-ACP synthase II (KAS II), chloroplastic isoform 1	2623	1695	564	402	526	ON175904
14	3-oxoacyl-ACP synthase III (KAS III), chloroplastic	1917	1215	404	457	245	ON175905
15	3-oxoacyl-ACP reductase 1 (FabG)	2151	867	288	312	972	ON175906
16	3-oxoacyl-ACP reductase 2 (FabG)	1672	909	302	485	278	ON175907
17	3-hydroxyacyl-ACP dehydratase (FabZ)	1157	675	224	265	217	ON175908
18	Enoyl-ACP reductase, chloroplastic (FabI)	1720	1176	391	252	292	ON175909
19	Stearoyl-ACP 9-desaturase 1 (SAD1), chloroplastic	1882	1191	396	225	466	ON175910
20	Stearoyl-ACP 9-desaturase 5 (SAD5), chloroplastic	1956	1170	389	206	580	ON175911
21	Stearoyl-ACP 9-desaturase 6 (SAD6), chloroplastic	1425	1197	398	56	172	ON175912
22	Long chain acyl-CoA synthetase 1 (LACS1)	2854	2016	671	307	531	ON175913
23	Long chain acyl-CoA synthetase 2 (LACS2)	2570	1986	661	349	235	ON175914
24	Long chain acyl-CoA synthetase 9, (LACS9) chloroplastic	2969	2085	694	606	278	ON175915
25	Oleoyl-ACP thioesterase 1, chloroplastic (FatA)	3544	1119	372	884	1541	ON175916
26	Acyl-ACP thioesterase ATL3, chloroplastic (FatB)	1070	615	204	112	343	ON175917
27	Glycerol-3-phosphate acyltransferase 3 (GPAT)	1949	1128	375	339	482	ON175918
28	Glycerol-3-phosphate acyltransferase, chloroplastic (GPAT)	1823	1353	450	270	200	ON175919
29	1-acyl-sn-glycerol-3-phosphate acyltransferase (AGPAT)	2033	897	298	2	1134	ON175920
30	1-acyl-sn-glycerol-3-phosphate acyltransferase 1, chloroplastic (AGPAT1)	1888	1083	360	147	658	ON175921
31	1-acyl-sn-glycerol-3-phosphate acyltransferase 2 (AGPAT2)	1788	1149	382	297	342	ON175922
32	1-acyl-sn-glycerol-3-phosphate acyltransferase 3 (AGPAT3)	1807	1143	380	200	464	ON175923
33	Phosphatidate phosphatase 1 (PAP1)	3757	2676	891	614	467	ON175924
34	Phosphatidylcholine:diacylglycerol cholinephosphotransferase 1 (AAPT1)	1402	813	270	239	350	ON175925
35	Delta(12)-fatty acid desaturase 2 (FAD2)	1827	1155	384	259	413	ON175926
36	Delta(12)-fatty acid conjugase (FADX)	1674	1200	399	133	341	ON175927
37	Phospholipid:diacylglycerol acyltransferase 1 (PDAT1)	2934	2004	667	558	372	ON175928
38	Diacylglycerol O-acyltransferase 1A (DGAT1A)	2191	1596	531	320	275	ON175929
39	Diacylglycerol O-acyltransferase 2 (DGAT2)	2625	966	321	199	1460	ON175930

Bitter gourd seed oil's medicinal and industrial applications are attributed to the conjugated fatty acid, mainly α-eleostearic acid. Plants with a negligible amount of conjugated fatty acids can also be metabolically engineered to produce a higher amount of these fatty acids by transferring the relevant genes. In Soybean and Arabidopsis, conjugated fatty acid content increased by 15 to 20% when delta-9 and delta-12 conjugase genes were expressed under seed-specific promoters^46^. In Arabidopsis, the accumulation of con-jugated fatty acids was further improved by expressing both fatty acid desaturase and delta-9 and delta-12 conjugase^47^. Developing bitter gourd and other plant varieties with a high amount of conjugated fatty acids will require identifying the genes necessary to divert diacylglycerol from triacylglycerol synthesis. Three enzymes, CDP-choline diacyl glycerol choline phosphotransferase (AAPT), fatty acid desaturase (FAD), and fatty acid conjugase (FADX), are required to synthesize α-eleostearic acid from diacylglycerol^48^. Several transcripts related to the biosynthesis of conjugated fatty acids were identified by analyzing the normalized cDNA library of seed tissues^43^. However, the identification of full-length genes was limited to diacylglycerol acyltransferase 1 and 2, which do not directly affect the production of conjugated fatty acid. In the current study, we identified full-length genes for all three enzymes, all of which were present as single-copy genes (Fig. 9, Table 4). These genes will help to improve the conjugated fatty acid content in bitter gourd and other high-yielding oilseed crops like brassica, sunflower, groundnut, and soybean. Though this has been demonstrated in Arabidopsis by co‐expressing FADX and DGAT genes from tung^49^, further research in important oilseed crops is required to achieve the scale of production needed for medicinal and industrial applications.

## Materials and methods

### Plant material

**Figure 10:**
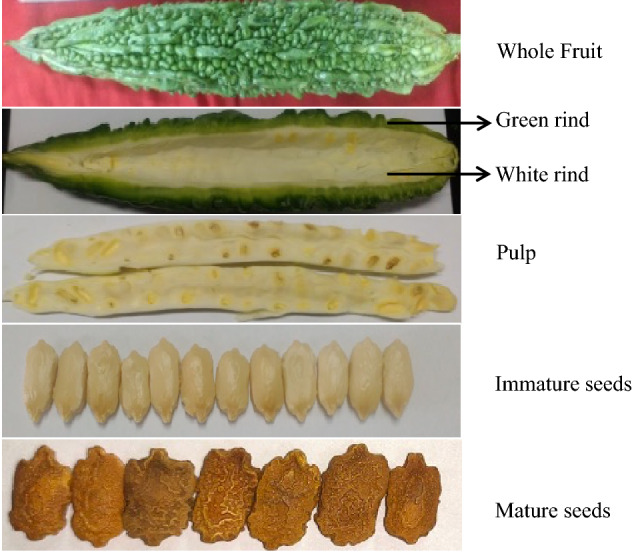
The five different tissues of bitter gourd fruits used for the transcriptome sequencing.

Seeds of Momordica charantia L. variety Co1 were obtained from Tamil Nadu Agricultural University, Coimbatore, Tamil Nadu, India. It is a cultivated variety and its fruits are usually long and dark green weighing upto 100–120 g. It was released for cultivation in 1978, and can yield up to 14 tonnes/ha. The plants were raised from the seeds in a greenhouse (23–25 ºC temperature, 16 h light, and 50% relative humidity). Green unripe fruits were cleaned with sterile distilled water, and dissected to separate green rind, white rind, pulp, and immature seeds. Mature seeds were collected from ripe fruits (Fig. 10). The separated tissues were flash-frozen under liquid nitrogen before storing in a freezer (-80 °C) until use. The plant studies were carried out in accordance with relevant institutional, national, and international guidelines and legislation.

### RNA isolation

RNA isolation from the different samples of *M.charantia* fruits was done using the Trizol reagent (Invitrogen). Frozen tissue sample of 100 mg was ground to fine powder under liquid nitrogen, 1 ml of Trizol was added, and the tissue suspension was transferred to a 1.5 ml centrifuge tube. Chloroform (0.2 volume) was added, mixed vigorously, and centrifuged at 12,000 rpm for 15 min at 4 °C. The aqueous phase was transferred to a fresh 1.5 ml centrifuge tube, and 0.7 volume of isopropanol was added. The content was mixed gently, centrifuged at 12,000 rpm for 10 min at 4 °C, and the supernatant was discarded. The pellet was washed by adding 500 µl of 70% ethanol, and centrifuging at 10,000 rpm for 5 min at room temperature. The supernatant was discarded, pellet was air-dried, and dissolved in nuclease-free water. The total RNA was checked in the gel, and treated DNase I (Qiagen, Germany) followed by column purification using RNeasy MinElute Cleanup Kit (Qiagen, Germany). The purified RNA was assessed using a spectrophotometer (Eppendorf, Germany), Qubit 2.0 fluorimeter (Invitrogen, USA), and Bioanalyzer (Agilent Technologies, USA). The RNA integrity number (RIN value) of RNA samples ranged from 7.3 to 9.3. The RNA samples with RIN values above eight were used for complementary DNA synthesis.

### Library preparation and sequencing

The sequencing library was prepared using one µg total RNA as a template using TruSeq mRNA Library Preparation Kit V2 (Illumina Inc, USA). Poly(A)-RNA was purified from total RNA using oligo-dT magnetic beads and fragmented before cDNA synthesis. The fragmented cDNAs were used as template for cDNA synthesis using SuperScript II reverse transcriptase (Invitrogen, USA). The first strand cDNA was converted to double strand cDNA and purified using AMPure XP beads. The purified cDNAs were end-repaired to generate blunt-ended cDNAs and single 'A' nucleotide tail was added to the 3' ends. These 'A' tails were complementary to the 'T' overhang in the adapters. Adapters were ligated to the cDNA ends for indexing and size selected for 420 bp fragments using AMPure XP beads. The size-selected cDNAs were enriched by PCR. The PCR-amplified library was purified, and analyzed in Bioanalyzer 2100 (Agilent Technologies, USA) using High Sensitivity DNA Chips to determine the insert size and yield. The DNA yield was normalized to 10 nM, and pooled for cluster generation in the flow cell. The libraries were subjected to 2 × 150 bp paired-end sequencing in the Illumina platform (Illumina, USA).

### De novo assembly and clustering

FastQC v0.11.2^27^ was used for the quality analysis of the reads. Removal of adaptor sequences and trimming was performed using Cut adapt v1.7.1^50^ and Sickle v1.33^51^. The high-quality (Q30 >) paired-end reads were assembled using the Trinity assembler with three modules^52^. Inchworm assembled unique transcripts using k-mer values. Chrysalis clustered related contigs and constructed de Bruijn graphs. Butterfly reconstructed full-length transcripts and distinct transcripts for splice isoforms. The redundancy among the assembled transcripts was reduced by clustering using CD-HIT v4.0^53^.

### Annotation of the transcripts

To perform functional annotation, de novo assembled transcripts of bitter gourd were analyzed using BLASTX algorithm plant non-redundant protein database at National Center for Biotechnology Information (NCBI). The annotations of the best hits were saved and subjected to gene ontology (GO) analysis using Blast2GO. Kyoto Encyclopedia of Genes and Genomes (KEGG) database was used, and the transcripts were mapped to different pathways^54,55^.

### Transcript quantification

The abundance of the transcripts in the bitter gourd fruit tissues was measured using RNA-Seq by expectation maximization (RSEM) tool. In RSEM, the RNA-Seq reads were aligned to the reference transcripts to estimate the transcript abundance^56^. The fragments per kilobase per million (FPKM) and transcripts per million (TPM) were calculated to estimate the transcript abundance.

### Identification of simple sequence repeats

Simple sequence repeats (SSRs) from bitter gourd were identified using the MISA tool^57^. The search parameters identified different repeat types and number repeat for each kind.

### Assessment of gene completeness

Completeness of the transcripts was estimated using Benchmarking Universal Single Copy Orthologs (BUSCO) analysis against a database of single-copy orthologous genes for plants (eudicotyledons_odb10). The BUSCO analysis identifies the conserved orthologs and helps to assess the completeness of transcripts^58^.

### Validation of transcript assembly

Ten assembled transcripts were selected at random and validated for the correctness of de novo assembly by reverse transcription PCR (RT-PCR) amplification of the transcripts. Transcript-specific primers were designed based on the sequence of the de novo assembled transcripts (Supplementary Table S1). cDNA was synthesized using oligo-dT primers, and individual transcripts were amplified using the respective transcript-specific primers. PCR amplification included initial denaturation at 95 ºC for 2 min, 35 cyles of denaturation at 95 ºC for 30 s, annealing (55–59 ºC) for 30 s, and extension at 72 ºC for 2 min, final extension at 72 ºC for 5 min, and storage at 4 ºC. The amplified products were analyzed by agarose gel electrophoresis.

### Analysis of gene expression

Five transcripts were selected at random and analyzed for gene expression. Tran-script-specific primers suitable for qRT-PCR were designed based on the sequence of the de novo assembled transcripts. Analysis of gene expression was performed using TB Green Premix Ex Taq II (Takara, Japan) and QuantStudio 5 (ThermoFisher Scientific, USA). As reported before, the serine/threonine phosphatase gene was used as an internal reference^59^. A negative control reaction without a template was included in all experiments done. The sequences of the primers designed for qRT-PCR analysis are given in Supplementary Table S2. Gene expression was analyzed using the individual efficiency corrected calculation method^60^.

## Conclusions

In the current study, we investigated the fruit transcriptome of bitter gourd, focusing on identifying full-length genes involved in seed oil biosynthesis. We identified 39 full-length genes coding for 23 enzymes and two carrier proteins involved in the biosynthesis of fatty acids, conjugated fatty acids, and triacylglycerol. We also identified several transcripts related to the biosynthesis of the compounds responsible for the bitterness and antidiabetic properties. The tissue-specific abundance of the transcripts reported here will form the basis for identifying tissue-specific promoters. The full-length genes, gene expression data, and SSR markers reported from this study are vital genomic resources for further research.

## Supplementary Information

Supplementary Information.

## Data Availability

Datasets used in this study were deposited in the SRA (https://www.ncbi.nlm.nih.gov/sra) under the SRA accession numbers SRR18210038 (immature seeds), SRR18210039 (pulp), SRR18210040 (mature seeds), SRR18210041 (white rind), and SRR18210042 (green rind). The full length oil biosynthesis genes were deposited in NCBI GenBank (Accession No: ON175892–ON175930).
