# Supplementary material for: Transcriptome analysis of five different tissues of bitter gourd (Momordica charantia L.) fruit identifies full-length genes involved in seed oil biosynthesis
Source: Sci Rep. 2022 Sep 13;12:15374. doi: 10.1038/s41598-022-19686-4 (PMC9470707; doi:10.1038/s41598-022-19686-4)
Supplement: Supplementary file 1 — Supplementary Information. [file 41598_2022_19686_MOESM1_ESM.zip › Supplementary file/Supplementary Figure legend.docx]

**Transcriptome analysis of five different tissues of bitter gourd (*Momordica charantia* L.) fruit identifies full-length genes involved in seed oil biosynthesis**

Kumar Ravichandiran and Madasamy Parani*

Department of Genetic Engineering, College of Engineering and Technology, Faculty of Engineering and Technology, SRM Institute of Science and Technology, SRM Nagar, Kattankulathur 603203, Chengalpattu, Tamil Nadu, India

*Author for correspondence: [paranim@srmist.edu.in](mailto:paranim@srmist.edu.in)

**Supplementary Figure legends**

**Supplementary Figure S1.** Gene completeness analysis of the assembled bitter gourd transcripts using BUSCO.

**Supplementary Figure S2.** Transcripts from bitter gourd fruit transcriptome mapped to terpenoid biosynthesis pathway (map00900) based on KEGG pathway analysis

**Supplementary Figure S3**. Transcripts from bitter gourd fruit transcriptome mapped to steroid biosynthesis pathway (map00100) based on KEGG pathway analysis
