# Supplementary figures and images for: Transcriptome analysis of five different tissues of bitter gourd (Momordica charantia L.) fruit identifies full-length genes involved in seed oil biosynthesis
Source: Sci Rep. 2022 Sep 13;12:15374. doi: 10.1038/s41598-022-19686-4 (PMC9470707; doi:10.1038/s41598-022-19686-4)

# BUSCO Assessment Results

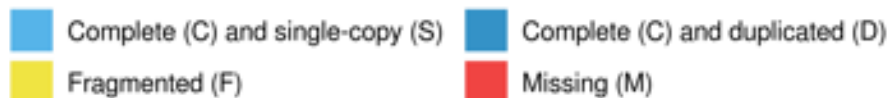

C:1997 [S:849, D:1148], F:72, M:52, n:2121

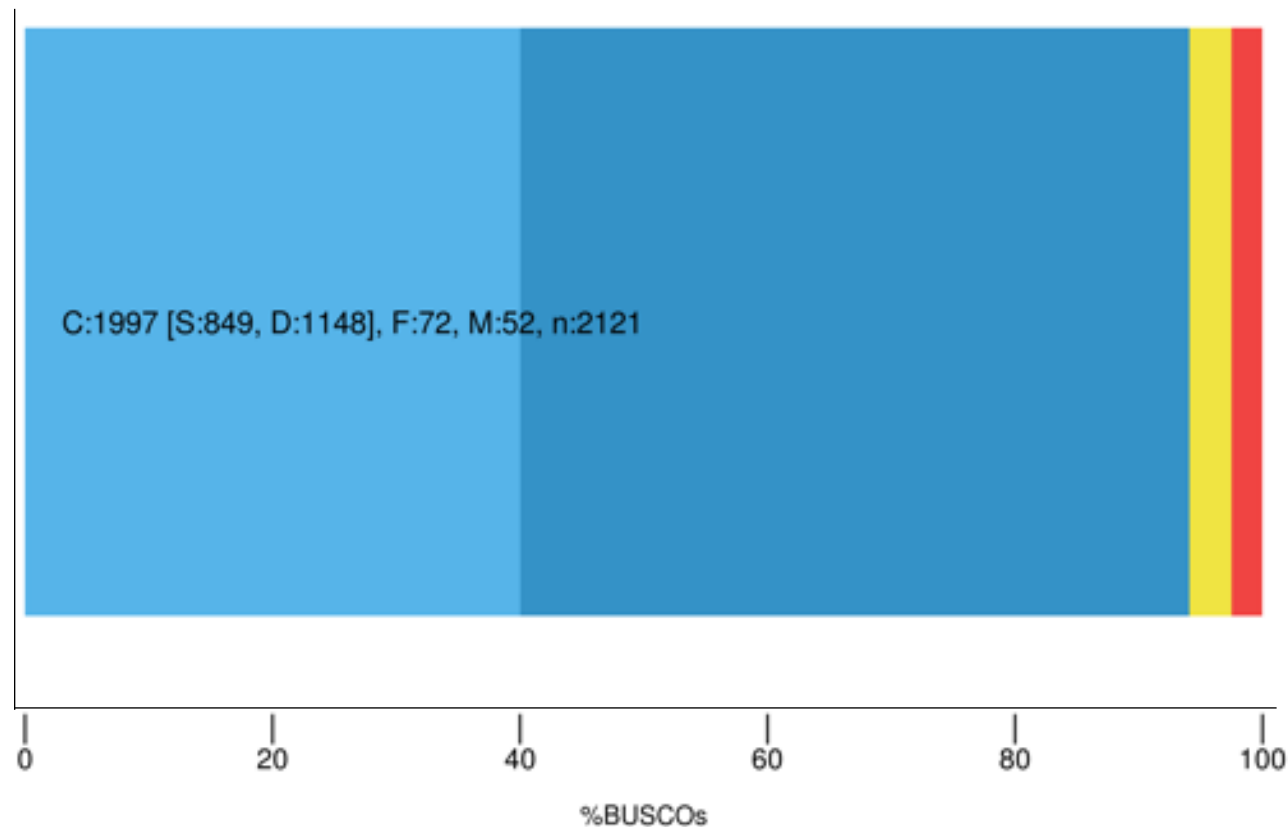

Supplement: Supplementary file 1 — Supplementary Information. [file 41598_2022_19686_MOESM1_ESM.zip › Supplementary file/Supplementary Figure S1 .pdf]

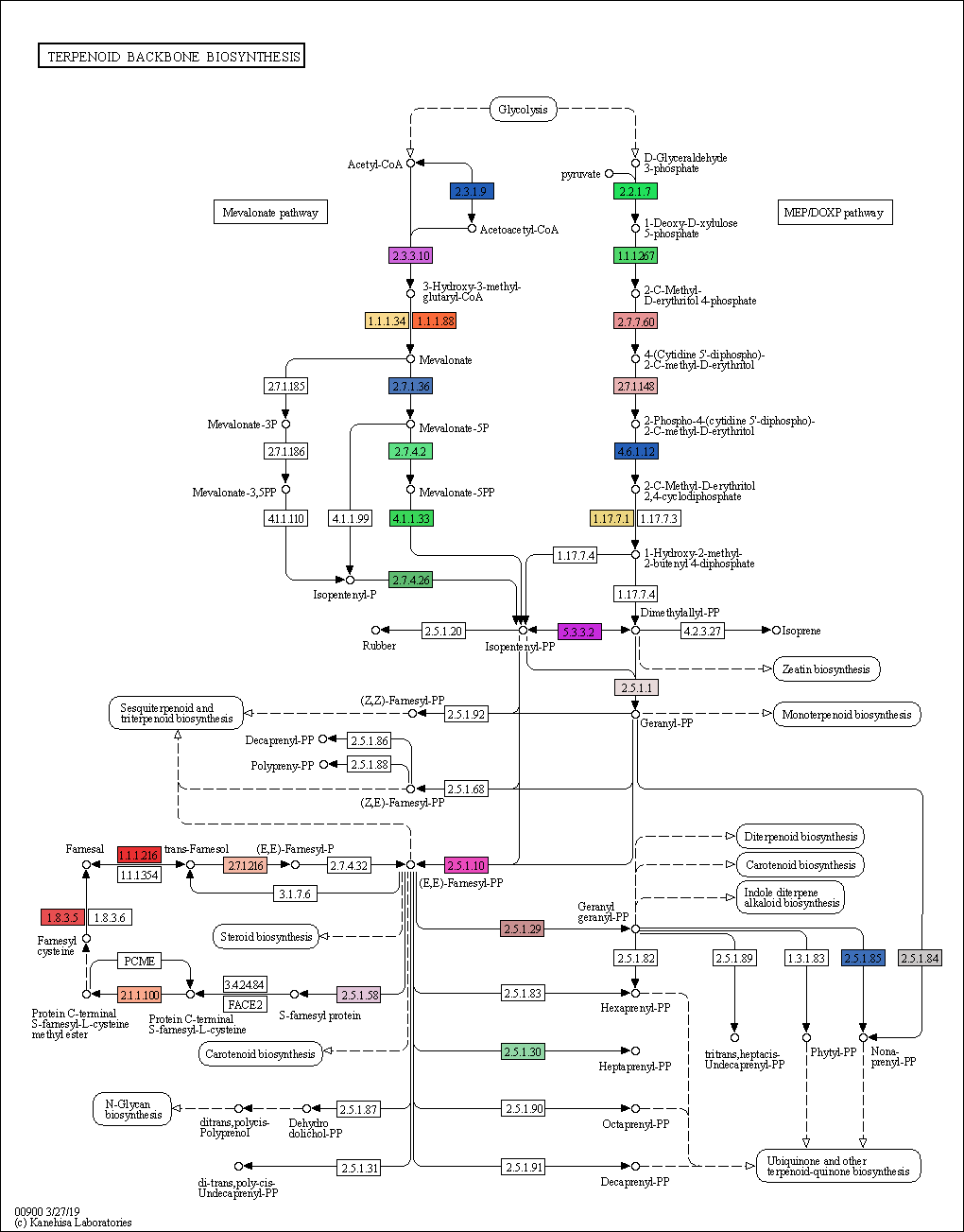

Supplement: Supplementary file 1 — Supplementary Information. [file 41598_2022_19686_MOESM1_ESM.zip › Supplementary file/Supplementary Figure S2.png]

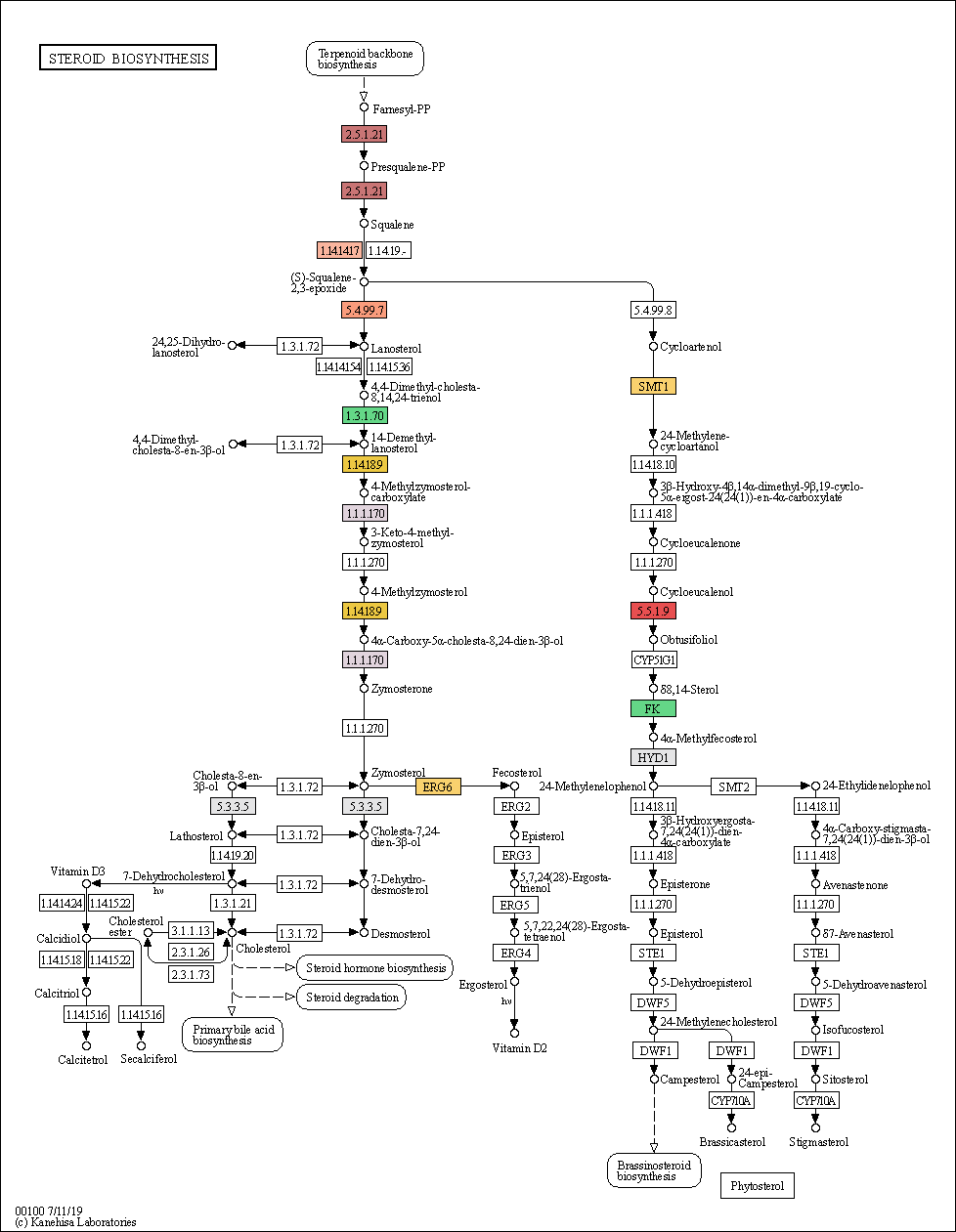

Supplement: Supplementary file 1 — Supplementary Information. [file 41598_2022_19686_MOESM1_ESM.zip › Supplementary file/Supplementary Figure S3.png]
